# Fatal Effects of Chloroform

**Published:** 1856-01

**Authors:** 


### EDITORIAL DEPARTMENT.

*Fatal Effects of Chloroform.*—We publish in another part of the present No. of the Journal an article on the manner in which death is occasioned by the inhalation of chloroform; also an account of a death recently produced in Edinburgh, by the use of this anæsthetic agent, and in the last Boston Medical and Surgical Journal, we find the following account of the death of a lady, produced by the use of the same agent. There are some surgical operations, the magnitude and importance of which, may, we do not deny, justify the use of chloroform, but, the extraction of a tooth is too simple to warrant the employment of so powerful an agent, and we do not think a dentist should permit himself to be prevailed upon to administer it for this purpose under any circumstances, except by the advice and under the direction of the family physician of the patient.—EDS.

